# “One Shot” Sample Evaluation of 22G, 22G upgraded, 21G and 19G needle for Endobronchial Ultrasound-EBUS-TBNA

**DOI:** 10.7150/jca.74022

**Published:** 2022-07-18

**Authors:** Rena Oikonomidou, Dimitris Petridis, Petros Alexidis, Dimitris Matthaios, Ioannis Boukovinas, Eleni Isidora Perdikouri, Sofie Baka, Wolfgang Hohenforst-Schmidt, Haidong Huang, Chong bai, Bojan Zaric, Lutz Freitag, Nikolaos Courcoutsakis, Marios Anemoulis, Christoforos Kosmidis, Christoforos Foroulis, Savas Petanidis, Vasilis Papadopoulos, Aris Ioannidis, Paul Zarogoulidis

**Affiliations:** 1 Health center Evosmos, Thessaloniki, Greece; 2 Department of Food Technology, School of Food Technology and Nutrition, Alexander Technological Educational Institute, Thessaloniki, Greece; 3 Radiotherapy Department, ``G. Papageorgiou`` University Hospital, Aristotle University of Thessaloniki, Thessaloniki, Greece; 4 Oncology Department, General Hospital of Rhodes, Rhodes, Greece; 5 Oncology Department, ``Bioclinic`` Private Hospital, Thessaloniki, Greece; 6 Oncology Department, General Hospital of Volos, Greece; 7 Oncology Department, ``Interbalkan`` European Medical Center, Thessaloniki, Greece; 8 Sana Clinic Group Franken, Department of Cardiology / Pulmonology / Intensive Care / Nephrology, ''Hof'' Clinics, University of Erlangen, Hof, Germany; 9 Department of Respiratory & Critical Care Medicine, Changhai Hospital, the Second Military Medical University, Shanghai, China; 10 Faculty of Medicine, University of Novi Sad, Institute for Pulmonary Diseases of Vojvodina, Novi Sad, Serbia.; 11 Department of Pulmonology, University Hospital Zurich, Rämistrasse 100, 8091, Zurich Switzerland; 12 Radiology Department, Democritus University of Thrace, Alexandroupolis, Greece; 13 Surgery Department, General Clinic Euromedica, Thessaloniki, Greece; 14 3rd Department of Surgery, ``AHEPA`` University Hospital, Aristotle University of Thessaloniki, Medical School, Thessaloniki, Greece; 15 Thoracic Surgery Department, ``AHEPA`` University Hospital, Aristotle University of Thessaloniki, Thessaloniki, Greece; 16 Department of Chemical Engineering, Aristotle University of Thessaloniki, Thessaloniki, Greece; 17 Oncology Department, University General Hospital of Thessaly, Thessaly, Greece; 18 Surgery Department, Genesis Private Clinic, Thessaloniki, Greece; 19 Pulmonary Oncology Department, ``Bioclinic`` Private Hospital, Thessaloniki, Greece

**Keywords:** lung cancer, biopsy, bronchoscopy, 19G needle, 21G needle, 22G needle, EBUS-TBNA, lymphnodes

## Abstract

**Background:** There are still diagnostic issues with lung cancer and mediastinum lymphadenopathy. Endobronchial ultrasound (EBUS) is a state of the art equipment for the diagnosis of lymphadenopathy and central lesions.

**Objective:** To investigate the sample size with one pass.

**Patients and Methods:** 248 Stage IV patients were included in our study. All patients had a CT of the thorax with either lymphadenopathy or lyphadenopathy plus pulmonary lesions. Patients had a biopsy with endobronchial ultrasound with 22G Mediglope, 22G Mediglope Sonotip, 21G Olympus and 19G Olympus needle. We collected information regarding the cancer type, cell block, tissue, age, sex, lesion size and needle type.

**Results:** The cancer type diagnosis was associated with the needle diameter. The number of cell-blocks were associated with the lesion size and needle diameter. Slices from the tissue and cell-blocks were again associated with the lesion size and needle diameter.

**Conclusion:** One pass is enough for cancer diagnosis, however; careful selection has to be made among patients regarding the needle diameter. In the case of lymphoma suspicion we should use 19G needle.

## Introduction

Lung cancer is usually diagnosed at advanced stage and therefore systemic treatment is necessary. There are several mathods to obtain biopsy samples either from peripheral lesions or central lesions. Regaring peripheral lesions we can use computed tomography guided biopsies and transthoracic ultrasound biopsies with convex probe ultrasound. Usually for these two methods we use 18G needles which aqcuire tissue 1. We can also use endoscopic techniques with radial endobronchial ultrasound, with or without the assistance of fluoroscopy 2-4. There are also novel electromagnetic navigation systems such as the superdimension^TM^ and the ARCHEMEDES^®^ 5. Regarding these techniques we can use 25G, 22G, 21G and 19G needles which aqcuire both tissue and cells. We also use biopsy forceps and cryobiopsy catheters. Another radiologic equipment for real time biopsy is the Cios Spin 3D by Siemens and other companies such as Philips 6-9. Regarding central lesions next to vessels we can use the convex probe endobronchial ultrasound (EBUS) from three different companies such as; PENTAX, OLYMPUS and FUJI. Through these endoscopes we can use 25G, 22G, 21G and 19G needles and small biopsy forceps 10-14. Moreover; regarding needle biopsies we use the cell block technique in order to create a paraffin block. In all these techniques we perform molecular investigation for epidermal growth factor (EGFR), anaplastic lymphoma kinase (ALK), Proto-oncogene tyrosine-protein kinase (ROS-1), proto-oncogene (BRAF) programmed death-ligand 1 (PD-L1) , part of the RAS/MAPK pathway (KRAS), abnormal neurotrophic tyrosine receptor kinase (NTRK), tyrosine-protein kinase Met (MET), proto-oncogene RET (RET), and erythroblastic oncogene B (HER2) 15-18. In inoperable patients it is absolutely necessary to have the molecular profile of the malignancy in order to administer targeted treatment. Elastography is another tool that has been investigated as a surrogate tool for initial diagnosis however; currently it is not widely used 19-21. In our current study we will evaluate four different needle types with EBUS-transbronchial needle aspiration (TBNA) 22G Mediglope, 22G Mediglope Sonotip, 21G Olympus and 19G Olympus needle for mediastinal lymphadenopathy. We will evaluate the diagnostic value of a single pass with the different needle types.

## Patients and Methods

**Figure 1:**
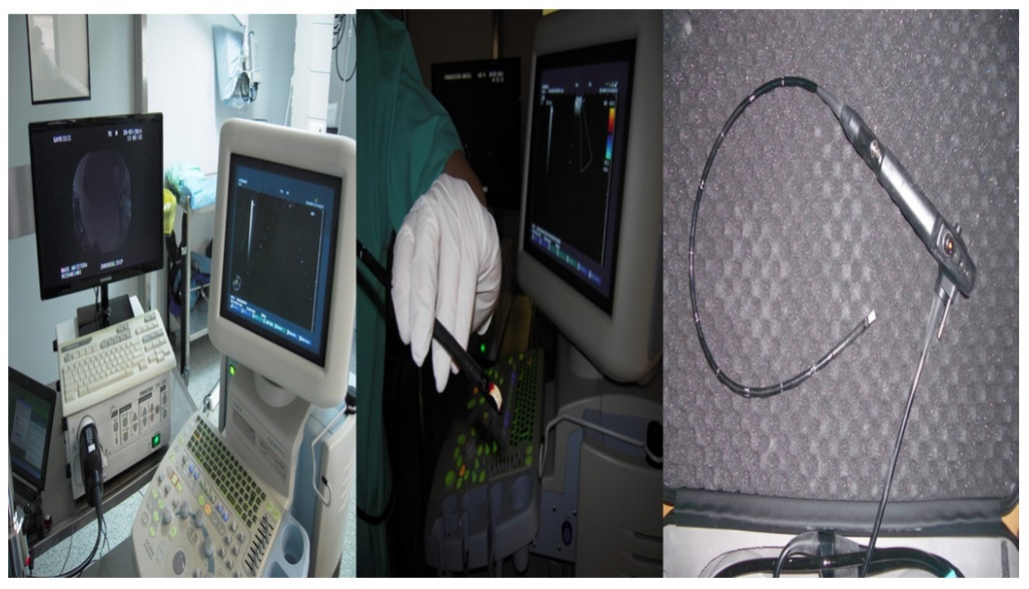
*PENTAX EBUS EB-1970UK*.

**Figure 2:**
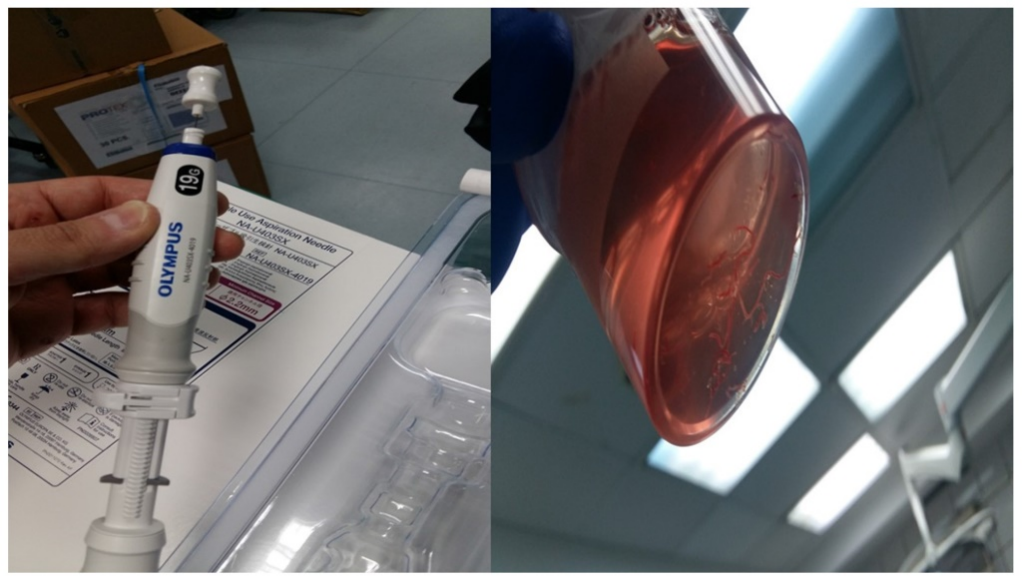
Left: 19G Olympus^®^ and Right: the sample which is tissue.

**Figure 3:**
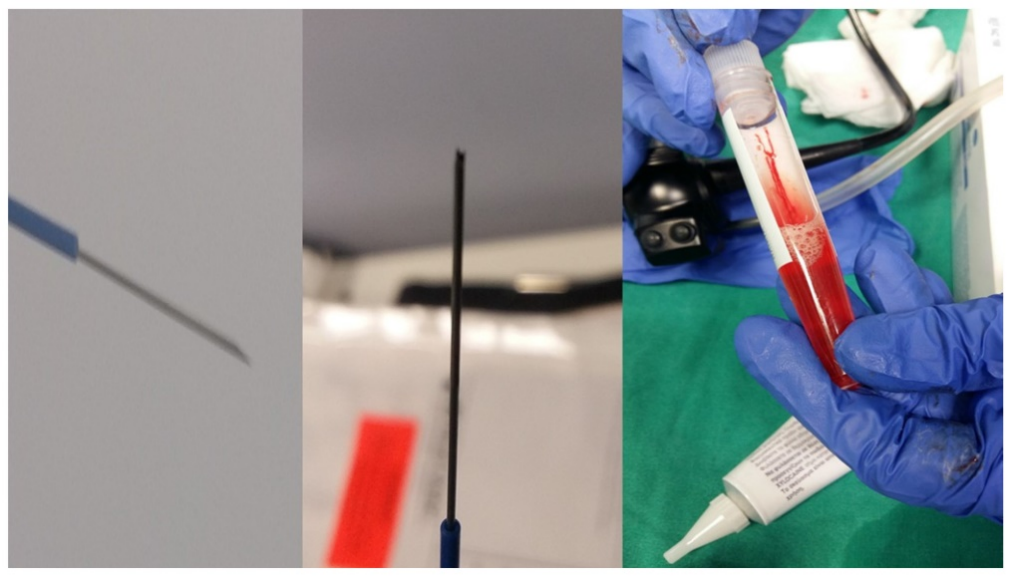
Left: 22G Mediglobe^®^, Middle: 22G Mediglobe^®^ upgraded needle and Right: the sample which is like a ``pus`` material.

Two hundred and fourty eight patients were included in our retrospective study that was approved by our investigational review board (IRB) 14/2019 3^rd^ University Surgery Department, AHEPA HOSPITAL, Thessaloniki, Greece. Inclusion criteria: 1) All patients were stage IV, 2) were ≥18years old and 3) had previously Positron emission tomography-CT (PET-CT). Moreover; 4) all patients were fit to undergo anesthesia for endoscopy, 5) performance status ECOG ≤3 and 6) oxygen saturation ≥60%. We performed endobronchial ultrasound (EBUS) in all our patients. All patients included had lymphadenopathy of the mediastinum with or without a lung cancer lesion inside the lung parenchyma. All hospitals included had the same equipment and needles. Our study was multicenter and all involved departments used a PENTAX EB-1970UK endobronchial system. We used the following needles: 1) 22G Mediglobe®, 22G Upgraded Mediglobe (MNediSonotip)^®^, 3) 21G Olympus^®^ and 4) 19G Olympus^®^. The difference between needles number 1 and 2 is the differentiation of the tip. **Figure 1-3.** Exclusion criteria: 1) Patients with lymphadenopathy that had previously been diagnosed with a cancer and had a high probability being diagnosed with a relapse disease, 2) had severe heart condition and were not able to receive anesthesia for endoscopy, 3) patients with ECOG ≥3, and 4) patients with oxygen saturation ≤60%. It has been observed in a previous study that although we have the same needle diameter needle type number 2 acquires more tissue sample 10. The data that we will present are from one single pass in an effort to present solid data regarding the number of passes which are necessary for diagnosis. In two hundred patients we had a diagnosis with a single pass. In two hundred and eight patients we performed next generation sequencing (NGS) for gene investigation.

### Statistical Analysis

#### Materials and Methods

The lesion size (cm) was considered as the key-variable. The lesion size potentially determines the use of different needle size and also the type of cancer. Moreover; the lesion size is associated with the number of slices, formation or not of cell blocks and tissue sample size. All aforesaid parameters were scheduled to predict the right needle size for every lesion. Thus, to relate the predictors with the response variable, a partition model was applied via JMP 14.3 (SAS Inc, 2017) statistical software. The partition algorithm repeatedly attempts all possible bifid splits of predictors in order to end up with the best decision tree model, conforming to some important assumptions, such as the least permitted number of 5 observations in the final nodes after data splitting. For categorical parameters the fitted value is a probability per level of each response at every split and the main goal is to maximize the difference between the two nodes of the split. For continuous predictors, such as the number of slices, the split creates a cutting value leading to two different nodes. The G^2^ statistic (likelihood-ratio chi-square) is used for categorical responses and lower values indicate a better fit.

To increase the validity of the splitting technique, the sample was randomly divided into two sets, the training, that is the part used to estimate model parameters (70% of patients) and the validation set (30%), useful for assessing the predictive ability of the model. Additionally, a random 10-fold cross-validation was attempted in the training sample and comparison was made between the total folded and the overall R^2^ coefficient of the best model determination. The reliability of data partitioning was checked by examining the confusion matrix produced by the training and validation set, which actually calls for misclassification rates.

## Results

Categories that prevail in each parameter of the study were male patients in a 3:2 ratio, lesion size 1-2 cm (41.9%), 19G Olympus needle (35.9%), nsclc cancer (51.6%) tissue material (62.9%) and present cell blocks (71.8%), **Table 1**.

**Figure 4:**
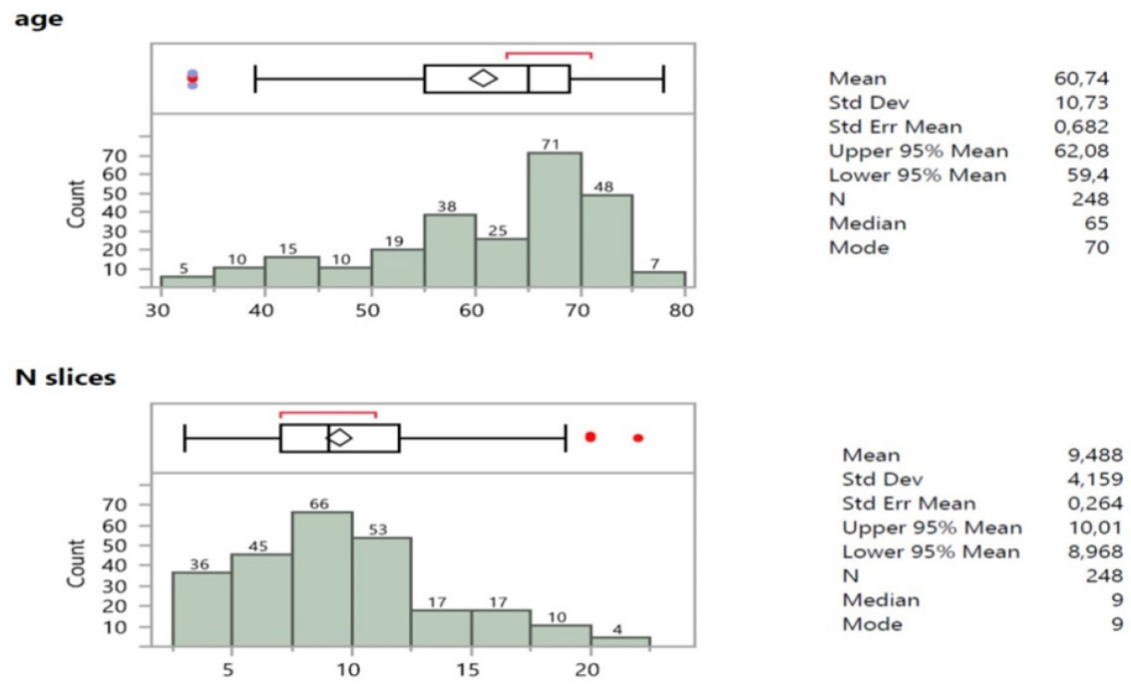
Summary statistics, box plot and size frequency distribution of age and number of slices of patients.

The age of patients was not normally distributed (**Figure 4**), mostly represented by ages >60 y.o., that is 60,9% (151*100/248). The number of cell block slices were skewed to the right in the size frequency distribution (**Figure 4**) revealing a median value and node of 9 slices.

The partition report of lesion size is shown in **Supplementary Figure 5**, in which successive splits led to 13 adequately classified nodes with different predictive category representatives.

This splitting diagram was judged quite efficient according to the statistical information shown in **Supplementary Figure 6**.

Thirteen splits gave the best solution of data partioning revealing the highest values of training and validation determined R-squares (0,700 and 0,698 respectively). Values close to 1 show a perfect fit of the model. Similar R^2^ results were obtained from the 10-folded and overall data consideration (0,681 and 0,750). Additional statistical measures, such as Nagelkerke R^2^ (0,905), RMSE (root mean squared error), and mean absolute deviations, ratify a valid partition model (the lower the values the better fit). The misclassification rates for the training and validation sets appear exceptionally low, down to 11,52% and 16,87% respectively, thus strongly supporting a reliable partioning. They are also explicitly depicted in the confusion matrices of training and validation sets as off-diagonal frequencies.

Most important splitting variables were in accordance, the cell blocks contributing 48,7%, cancer type (19,92%), number of slices (15,78%), needle type (11,08%) and tissue material (4,5%).

The decision tree in **Supplementary Figure 5** is more interpretably manifested in the leaf report of **Supplementary Figure 7**. A single pathway (only one split) determines the absence of cell blocks in 46 patients and corresponds uniquely (probability 0,986) to the smallest lesion size (0,5-1 cm). The intermediate size (1-2 cm) is best described by four pathways with probability contribution >0,900 and also by three pathways with probability >0,800. The largest size (>2 cm) is best explained by one pathway (0,958), a second (0,8475) and a third one (0,768) 22, 23.

## Discussion

The endobronchial ultrasound has the limitation until today where fine needle aspiration (FNA) needles are used for biopsy. Current needles are 22G, 21G and 19G. The best method to store and evaluate the sample from the 22G, 21G and 19G needles is the cytolite fluid and cell block technique 10. Regarding the 19Gneedle we can use formole since we aqcuire more tissue sample and we can spare the cells from the biopsy. A major problem in the biopsy setting is the cost of the 19G needle and therefore we use it mostly in cases where lymphoma is suspected. Rapid on site evaluation (ROSE) is a techique that can be used for the evalaution of our sample on site during the biopsy procedure. The sample can be evaluated by a chest physician with the proper training and from a cytologist. In has been observed that the minimum number of passes necessary for the ROSE technique are two 24. It has been previously investigated how many passes are enough for lung cancer diagnosis, and how many passes are necessary in order to have enough sample size for next generation sequencing 25. Moreover; it has been previously observed that it is not only the needle size that matters regarding the sample size but also the needle tip shape 10. Also, most of the genes necessary for lung cancer treatment can be investigated from cell-blocks and smears 26. In our study we focused on the sample size of one single biopsy in different patients with different needle FNA needles from lymphnodes. It was observed as anticipated that lesions of 1≤cm had less cell blocks due to the small size of the lesion and 19G needle was used less for these lesions due to the limitations of the size. In most patients cell blocks were created (178/248). The number of slices from cell blocks were associated with the size of the lesion. The larger the lesion size ≥1cm the larger the number of slices. 19G needle had tissue instead of cell blocks, although cell-blocks could be created in some cases, tissue is considered the best material for diagnosis and we provided only this information. 22G needles and 21G needle provided only smear samples containing cells and tissue fragments. 19G needles were used mostly when lesions were ≥1cm for safety reasons and the diagnosis was strongly associated with lymphomas. There was no major difference regarding the number of slices between needles 22G and 22G upgraded as observed in our previous study 10.

## Conclusion

Therefore we conclude that a single pass is enough to make a diagnosis for lymphadenopathy, however; 19G needle should be used for lesions ≥1cm for safety reasons and in the case of suspected lymphoma. Regarding the passes that are necessary for next generation sequensing (NGS), at least two passes are necessary.

## Supplementary Material

Supplementary figures.

## Figures and Tables

**Table 1:** Numerical and percentage tabulation of the parameters under study from a sample of 248 patients.

Parameter	code	N	%
**gender**			
male	1	153	61.7
female	2	95	38.3
			
**lesion size (cm)**			
0.5-1.0	1	90	36.3
1.0-2.0	2	104	41.9
>2	3	54	21.8
			
**needle type**			
22GMedi	1	49	19.8
22GMNedi	2	54	21.8
21Golympus	3	56	22.6
19Golympus	4	89	35.9
			
**cancer type**			
nsclc	1	128	51.6
sclc	2	45	18.1
metastatic	3	28	11.3
non-hoghkin	4	33	13.3
Hoghkin	5	14	5.6
			
**tissue**			
no	0	92	37.1
yes	1	156	62.9
			
**cell blocks**			
no	0	70	28.2
yes	1	178	71.8
