# Supplementary material for: “One Shot” Sample Evaluation of 22G, 22G upgraded, 21G and 19G needle for Endobronchial Ultrasound-EBUS-TBNA
Source: J Cancer. 2022 Jul 18;13(10):2982–7. doi: 10.7150/jca.74022 (PMC9414030; doi:10.7150/jca.74022)
Supplement: Supplementary file 1 — Supplementary figures. [file jcav13p2982s1.pdf]

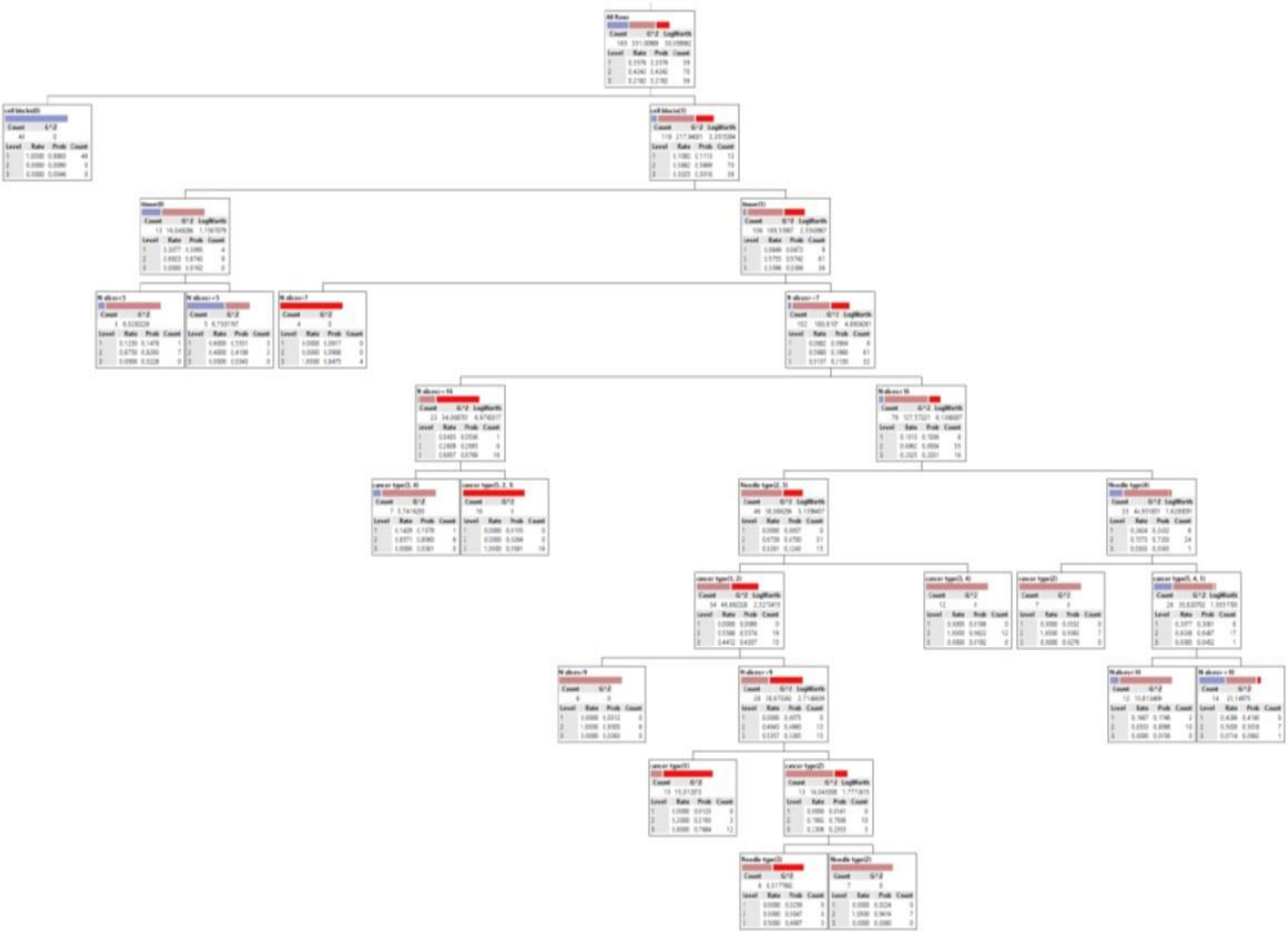

Split History

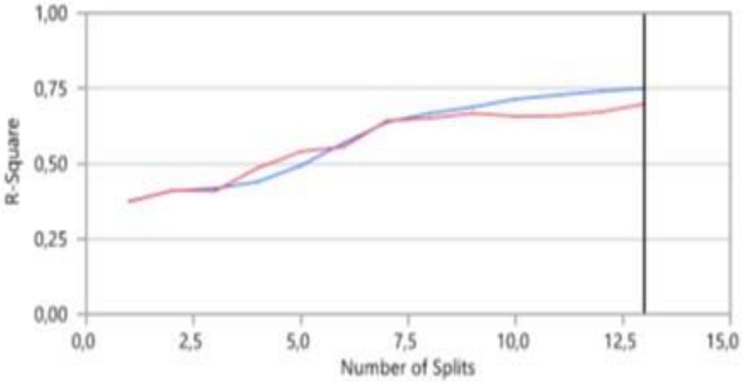

Validation Data in Red
Training set in blue

Fit Details

	RSquare	N	Number of Splits
Training	0,750	165	13
Validation (30%)	0,698	83	

Crossvalidation

k-fold	-2LogLike	RSquare
10 Folded	111,817273	0,6814
Overall	77,7931251	0,7501

Measure	Training	Validation	Definition
Entropy RSquare	0,7501	0,6982	1-Loglike(model)/Loglike(0)
Nagelkerke RSquare	0,9051	0,8783	(1-(L(0)/L(model))^(2/n))/(1-L(0)^(2/n))
Mean -Log p	0,2658	0,3214	∑ -Log(ρ[j])/n
RMSE	0,2889	0,3257	√ ∑(y[j]-ρ[j])²/n
Mean Abs Dev	0,1808	0,2172	∑ |y[j]-ρ[j]|/n
Misclassification Rate	0,1152	0,1687	∑ (ρ[j]≠ρMax)/n
N	165	83	n

Confusion Matrix

Training

Actual	Predicted Count		
lesion size (cm)	1	2	3
1	49	10	0
2	2	65	3
3	0	4	32

Validation

Actual	Predicted Count		
lesion size (cm)	1	2	3
1	25	6	0
2	2	27	5
3	0	1	17

Column Contributions

Term	Number of Splits	G^2	Portion
cell blocks	1	133,06988	0,4870
cancer type	4	54,4381518	0,1992
N slices	5	43,1144779	0,1578
Needle type	2	30,2616018	0,1108
tissue	1	12,332654	0,0451

Response probabilities

Leaf Label	1	2	3
cell blocks(0)	0,9863	0,0090	0,0046
cell blocks(1)&tissue(0)&N slices<5	0,1478	0,8293	0,0228
cell blocks(1)&tissue(0)&N slices>=5	0,5551	0,4106	0,0343
cell blocks(1)&tissue(1)&N slices<7	0,0617	0,0908	0,8475
cell blocks(1)&tissue(1)&N slices>=7&N slices>=16&cancer type(3, 4)	0,1579	0,8060	0,0361
cell blocks(1)&tissue(1)&N slices>=7&N slices>=16&cancer type(5, 2, 1)	0,0155	0,0264	0,9581
cell blocks(1)&tissue(1)&N slices>=7&N slices<16&Needle type(2, 3)&cancer type(1, 2)&N slices<9	0,0312	0,9305	0,0383
cell blocks(1)&tissue(1)&N slices>=7&N slices<16&Needle type(2, 3)^&N slices>=9&cancer type(1)	0,0123	0,2193	0,7684
cell blocks(1)&tissue(1)&N slices>=7&N slices<16^^&N slices>=9&cancer type(2)&Needle type(3)	0,0256	0,5047	0,4697
cell blocks(1)&tissue(1)&N slices>=7&N slices<16^^&N slices>=9&cancer type(2)&Needle type(2)	0,0224	0,9416	0,0360
cell blocks(1)&tissue(1)&N slices>=7&N slices<16&Needle type(2, 3)&cancer type(3, 4)	0,0186	0,9622	0,0192
cell blocks(1)&tissue(1)&N slices>=7&N slices<16&Needle type(4)&cancer type(2)	0,0332	0,9392	0,0276
cell blocks(1)&tissue(1)&N slices>=7&N slices<16&Needle type(4)&cancer type(5, 4, 1)&N slices<10	0,1746	0,8098	0,0156
cell blocks(1)&tissue(1)&N slices>=7&N slices<16&Needle type(4)&cancer type(5, 4, 1)&N slices>=10	0,4180	0,5018	0,0802

Response counts

Leaf Label	1	2	3
cell blocks(0)	46	0	0
cell blocks(1)&tissue(0)&N slices<5	1	7	0
cell blocks(1)&tissue(0)&N slices>=5	3	2	0
cell blocks(1)&tissue(1)&N slices<7	0	0	4
cell blocks(1)&tissue(1)&N slices>=7&N slices>=16&cancer type(3, 4)	1	6	0
cell blocks(1)&tissue(1)&N slices>=7&N slices>=16&cancer type(5, 2, 1)	0	0	16
cell blocks(1)&tissue(1)&N slices>=7&N slices<16&Needle type(2, 3)&cancer type(1, 2)&N slices<9	0	6	0
cell blocks(1)&tissue(1)&N slices>=7&N slices<16&Needle type(2, 3)^&N slices>=9&cancer type(1)	0	3	12
cell blocks(1)&tissue(1)&N slices>=7&N slices<16^^&N slices>=9&cancer type(2)&Needle type(3)	0	3	3
cell blocks(1)&tissue(1)&N slices>=7&N slices<16^^&N slices>=9&cancer type(2)&Needle type(2)	0	7	0
cell blocks(1)&tissue(1)&N slices>=7&N slices<16&Needle type(2, 3)&cancer type(3, 4)	0	12	0
cell blocks(1)&tissue(1)&N slices>=7&N slices<16&Needle type(4)&cancer type(2)	0	7	0
cell blocks(1)&tissue(1)&N slices>=7&N slices<16&Needle type(4)&cancer type(5, 4, 1)&N slices<10	2	10	0
cell blocks(1)&tissue(1)&N slices>=7&N slices<16&Needle type(4)&cancer type(5, 4, 1)&N slices>=10	6	7	1

Legends to the figures

Figure 5. Partition report for lesion size. Each terminal node informs for the number of counts, total and particular per category level, the candidate G^2 statistic corresponding to a logWorth value ($-\log_{10}$(p value)) and the probability fit contribution per level. Zero G^2 values signal a perfect division.

Figure 6. Statistical information on the validity and reliability of the partition modeling.

Figure 7. Leaf report of the 13 pathways according to the decision tree of the study, in response probabilities and numbers of patients.
